# Notes and News

**Published:** 1891-12-05

**Authors:** 


### NOTES AND NEWS.

Collected and Communicated.

The annual exhibition of food and cookery was held last week at the Agricultural Hall, Islington, N. The principal articles on view were beef preparations, pastry flours, cocoa, custard and baking powders, stoves, and such well-known manufactures as Bovril, Florador, and Coombe's Eureka pastry-flour, the latter excellent preparation having been awarded the gold medal last year at the same exhibition. As was stated in our review on July 25th of this year, "The flour is of excellent quality, and particularly well suited for making light and digestible pastry." Samples are offered to doctors, matrons, and nurses who care to write for them to 4, Stamford Street, Nottingham. Another exhibit of interest was that of Messrs. Phillips and Co., of Clerkenwell Road, who supply some of our largest institutions with chocolate, tea, and other groceries. Daily lectures on artistic cookery were given by Miss Kelman and Mrs. Thwaites, their object being the promotion of good cooking and the economic use of foods.

At the triennial festival dinner in connection with Charing Cross Hospital held at the Hotel Metropole, the Lord Mayor presided, and there was a large and influential attendance. The Council, in issuing their customary appeal on the occasion, expressed regret that they had to report that the past three years had not been a period of prosperity, a fact which was mainly due to a great falling off in the amount received from legacies. Concurrently with this, the number of patients had increased. Owing to the decrease of receipts the Council had had to draw on the savings of more prosperous years, until a sum of £2,000 only was now left; and at the present time there was a sum of £6,000 due to the bankers. An admirable site for a convalescent home had been acquired at Clacton-on-Sea through the generosity of a Governor; but £8,000 was still required to complete the buildings. Lastly, the necessity for rebuilding and enlarging the present quarters of the nursing staff became more earnest every day; and the Council, therefore, made a strong appeal for the funds necessary to meet all these demands, for which a sum of £30,000 was required. In the course of the evening it was intimated by the Lord Mayor that Mr. J. Passmore Edwards had addressed the hospital authorities offering to build and furnish solely at his own expense, a convalescent home with accommodation for 50 beds, enclosing a cheque for £5,000, and undertaking to provide for the remainder of the estimated cost of the home when its foundation stone had been laid. The only condition which Mr. Edwards attached to the gift was that the convalescent home should remain, in perpetuity, under the control of the Governors and Council of the hospital. Donations and subscriptions were announced during the evening to the amount of £7,576 (including Mr. Edwards's donation). Since the last festival the extension of the medical school has been completed and the nursing system has been thoroughly organised and developed.

The Treasurer of the Royal Hospital for Diseases of the Chest makes an earnest appeal for public support for the institution. It is most desirable to open, at as early a date as possible, the two remaining empty wards (containing thirty beds), which are lying idle through want of funds, and for which there is pressing need; but this cannot be accomplished until the income from annual subscriptions (£1,600) has been considerably augmented. Many subscribers, in recognition of the good work being done, have already increased, and in some cases doubled, their annual subscriptions, but further annual subscribers are urgently needed, in order to enable the Hospital to carry on and extend its beneficent sphere of action. The Hospital—the oldest of its kind in Europe—was founded in 1814, since which date it has carried on its humane work uninterruptedly. The time has unfortunately arrived when, with the available beds, it is quite impossible to receive all the poor who apply for admission, and the opening of one of the empty wards for the reception of adult patients, and of the other for the treatment of children, would prove a boon of inestimable value to hundreds of the suffering poor, whose condition demands immediate treatment and care.

The opening of the new building, in Templemore Avenue, of the Ulster Hospital for Children and Women constituted one of the most interesting of the many charitable functions that have recently taken place in the neighbourhood of Belfast. The hospital which has changed its location—certainly for the better—has for nearly twenty years discharged its duties in the centre of Belfast with exceptional success. We cannot doubt that the inhabitants of that populous neighbourhood, in the midst of which the hospital is now situated, will quickly come to appreciate the importance of the institution. Mr. J. Blakiston Houston, V.L., who, in the presence of a large and distinguished company, including the Countess of Shaftesbury, Lady Mildred, and Lady Violet Ashley, opened the building, called attention to many of the advantages likely to result from the removal of the hospital to the County Down side of the river. Dr. Dill said that, in respect of the work which had been done in the small hospital in Fisherwick Place, he might say that the medical staff had operated so far in such a way as to carry the blue ribbon in respect of certain operations, in which they were proud to say they had one hundred per cent. of recoveries.

At the Quarterly Court of the London Hospital, on Wednesday, the old question of the nursing arrangements was again brought up. Mr. Robert Hunter and Mr. Costelloe made admirable speeches, but seemed entirely ignorant of the fact that they were only repeating old charges which have long been refuted. It is noticeable that it is always those who have least acquaintance with the hospital, who have seldom been within its walls, who have given but little aid to its work, who are keenest in finding faults. The attacking party had secured a new recruit in the person of Miss D. Scrumgeour, once for three months a probationer in the wards. She was of opinion that the nurses and staff-nurses should have three weeks' holiday yearly, to which the Court heartily concurred; but she was also of opinion that this could be secured for one hundred pounds a year—arithmetic which was met by smiles and silence, and by 34 votes to 15, the matter was referred back to the House Committee. Too great disapprobation cannot be expressed for the perpetual "nagging" of the opposition; the Governors are quite strong enough to hold their own by overwhelming majorities whenever a question is put to the vote, but the mud-throwing has had the effect of draining the coffers of the institution, which is now so poor that with the best will in the world they cannot afford large improvements at present. The Hunters have damaged the cause of the nurses whom they professedly champion. We sincerely wish Mr. Costelloe and Mr. Hunter would take the opinion of nurses of long standing, and try and learn the practical points which are at issue; and then help the hospital to build extra rooms, &c., instead of injuring an old and excellent charity.

